# Regulation of human brown adipose tissue by adenosine and A_2A_ receptors – studies with [^15^O]H_2_O and [^11^C]TMSX PET/CT

**DOI:** 10.1007/s00259-018-4120-2

**Published:** 2018-08-13

**Authors:** Minna Lahesmaa, Vesa Oikonen, Semi Helin, Pauliina Luoto, Mueez U Din, Alexander Pfeifer, Pirjo Nuutila, Kirsi A. Virtanen

**Affiliations:** 1 Turku PET Centre, University of Turku, P.O. Box 52, FI-20520 Turku, Finland; 2 Turku PET Centre, Turku University Hospital, Turku, Finland; 3 Institute of Pharmacology and Toxicology, University of Bonn, Bonn, Germany; 4 Department of Endocrinology, Turku University Hospital, Turku, Finland

**Keywords:** Brown adipose tissue, Adenosine, A_2A_ receptor, Perfusion, Physiology, Positron emission tomography, [^11^C]TMSX, [^15^O]H_2_O

## Abstract

**Purpose:**

Brown adipose tissue (BAT) has emerged as a potential target to combat obesity and diabetes, but novel strategies to activate BAT are needed. Adenosine and A_2A_ receptor (A2AR) agonism activate BAT in rodents, and endogenous adenosine is released locally in BAT as a by-product of noradrenaline, but physiological data from humans is lacking. The purpose of this pilot study was to investigate the effects of exogenous adenosine on human BAT perfusion, and to determine the density of A2ARs in human BAT in vivo for the first time, using PET/CT imaging.

**Methods:**

Healthy, lean men (*n* = 10) participated in PET/CT imaging with two radioligands. Perfusion of BAT, white adipose tissue (WAT) and muscle was quantified with [^15^O]H_2_O at baseline, during cold exposure and during intravenous administration of adenosine. A2AR density of the tissues was quantified with [^11^C]TMSX at baseline and during cold exposure.

**Results:**

Adenosine increased the perfusion of BAT even more than cold exposure (baseline 8.3 ± 4.5, cold 19.6 ± 9.3, adenosine 28.6 ± 7.9 ml/100 g/min, *p* < 0.01). Distribution volume of [^11^C]TMSX in BAT was significantly lower during cold exposure compared to baseline. In cold, low [^11^C]TMSX binding coincided with high concentrations of noradrenaline.

**Conclusions:**

Adenosine administration caused a maximal perfusion effect in human supraclavicular BAT, indicating increased oxidative metabolism. Cold exposure increased noradrenaline concentrations and decreased the density of A2AR available for radioligand binding in BAT, suggesting augmented release of endogenous adenosine. Our results show that adenosine and A2AR are relevant for activation of human BAT, and A2AR provides a future target for enhancing BAT metabolism.

**Electronic supplementary material:**

The online version of this article (10.1007/s00259-018-4120-2) contains supplementary material, which is available to authorized users.

## Introduction

Brown adipose tissue (BAT) is involved in the metabolism and energy expenditure (EE) of humans. Instead of storing energy like white adipose tissue (WAT), BAT consumes calories and releases heat via non-shivering thermogenesis [1]. There is increasing evidence that enhanced BAT metabolism can improve systemic health in humans by utilizing glucose and lipids from the circulation [2–4]. Vast research is focusing on understanding regulatory factors involved in BAT metabolism, aiming to find new pharmacological approaches to activate thermogenesis and increase EE. This could serve as a potential new treatment strategy against the increasing epidemic of obesity and type 2 diabetes [5].

Adenosine is one important extracellular signaling molecule, which exerts a range of responses in different tissues, including adipose tissue. The effects of adenosine are mediated via four G protein-coupled receptor subtypes A_1_, A_2A_, A_2B_ and A_3_, [6] which all play a role in metabolism. Adenosine is significant in the pathophysiology of diabetes as it regulates proliferation and regeneration of β cells, controls cell responsiveness to insulin in adipose tissue, muscle and liver, and indirectly mediates inflammatory responses in these tissues [7]. It is also important for the normal physiology of BAT. Specifically, adenosine receptor A_2A_ (A2AR) signaling is required for full physiological activation of BAT, and selective activation of A2AR in WAT can induce transdifferentiation of white adipocytes towards a metabolically active brown phenotype [8]. A2AR agonists can also improve glucose homeostasis and adipose tissue inflammation in obese mice [9]. Therefore, modulating the adenosinergic system could be one potential way to activate BAT pharmacologically.

Although there is substantial preclinical evidence of the importance of adenosine for BAT function, the physiological significance in human BAT is still unexplored. Positron emission tomography (PET) combined with computed tomography (CT) offers a versatile tool for imaging phenomena in humans during different physiological states or drug administration. The objectives of this study were, firstly, to investigate the effects of adenosine on human BAT perfusion and, secondly, to quantify the density of A2AR in BAT in different physiological states. Radiowater, [^15^O]H_2_O, is commonly used to investigate tissue perfusion, and BAT perfusion is directly associated with oxygen consumption and EE [10–12]. [^15^O]H_2_O-PET imaging during adenosine administration has previously been used to study human skeletal muscle, cardiovascular function and WAT [13–15], but not BAT. Furthermore, previous studies have shown that A2AR is abundant in BAT of rodents [8], but information about A2AR in human BAT in vivo is still lacking. The PET radioligand [^11^C]TMSX binds specifically to A2AR, providing a tool to study changes in receptor physiology and occupancy. This method has previously been used to study the brain [16–18] and skeletal or cardiac muscle [19–21], but not adipose tissue.

We therefore designed a clinical study addressing these issues. In healthy, lean men we quantified BAT perfusion at baseline during adenosine stimulus and during cold exposure when BAT is physiologically activated. Additionally, we measured the density of A2AR in human BAT at baseline and during cold exposure.

## Materials and methods

### Study subjects

Ten male subjects were recruited to this study using electronic bulletin boards, and all provided written informed consent. The subjects were all healthy Caucasian men with an average age of 25 years (range 18–42 years). Subjects were determined healthy by clinical examination, blood tests and anthropometric measurements, and exclusion criteria included any significant chronic disease (e.g. asthma, diabetes, thyroid disease and cardiovascular disease), regular use of any medication and use of tobacco or nicotine products. The study protocol was reviewed and approved by the Ethics Committee of the Hospital District of Southwest Finland and was conducted according to the principles of the Declaration of Helsinki (Clinical trials ID NCT03327168).

### Study design

**Fig. 1:**
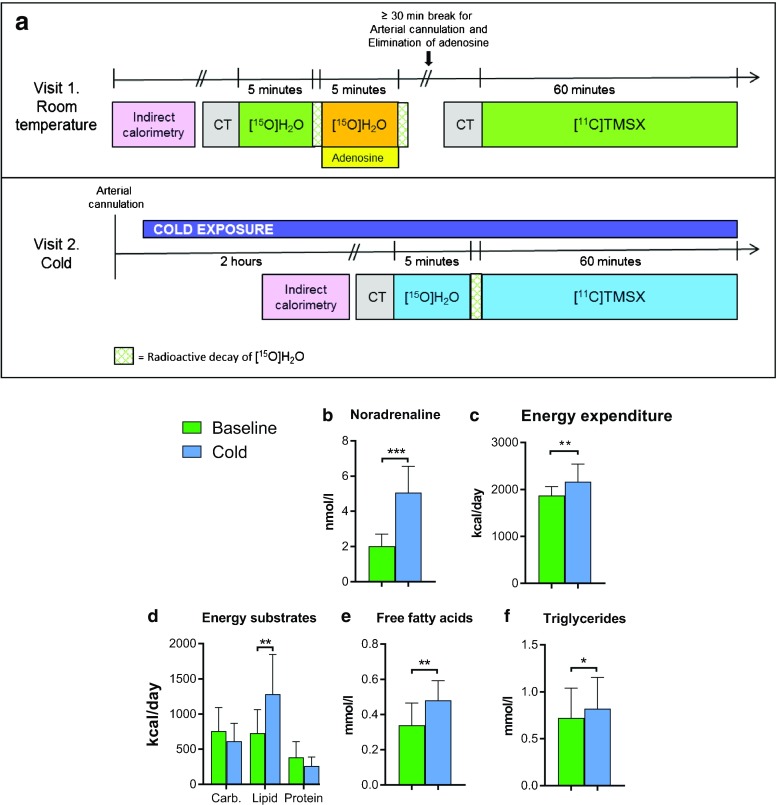
**A**. Diagram of the PET/CT imaging protocol. Each subject (*n* = 10) participated in imaging on 2 days with [^15^O]H_2_O and [^11^C]TMSX radioligands. **B**. Plasma noradrenaline (NA) values, **C**. energy expenditure (EE), **D**. the utilization of lipids as energy substrate, **E**. plasma-free fatty acids (FFAs) and **F**. plasma triglycerides increased during cold exposure. (Carb. = carbohydrates)

PET/CT scans were performed on two separate days in a random order, once in room-temperature conditions and once during controlled cold exposure (Fig. 1). Perfusion of BAT, WAT and muscle were measured using [^15^O]H_2_O-PET/CT in three different conditions: at baseline, during intravenous infusion of adenosine and during controlled cold exposure. Adenosine A2AR in BAT, WAT and muscle were quantified with [^11^C]TMSX-PET/CT twice: at baseline and during cold exposure. By design, the administered adenosine was fully eliminated from the body (during a ≥ 30-min break), before the baseline [^11^C]TMSX scan. Before imaging, the study subjects fasted overnight and avoided caffeine and strenuous exercise for a minimum of 24 h.

### Cooling protocol

A standardized cooling protocol was used on one scanning day. The subject lay in a supine position between cooling blankets (Blanketrol III, Cincinnati Sub-Zero, Cincinnati, OH, USA) with chilled flowing water for 2 h before the scan and during the PET/CT scan. The temperature of the blanket was determined and adjusted individually to maintain a subjectively cold temperature but avoid muscle shivering (average temperature 16.2 ± 1.6 degrees). All subjects reported cold sensation before and during scans. Electrocardiography was monitored during the cooling protocol and blood samples were acquired before and after cooling. Indirect calorimetry was performed using a Deltatrac II Datex-Ohmeda monitor at baseline and during cold conditions to measure whole-body EE according to the Weir equation [22] as previously described [10].

### PET imaging and analysis

PET radiotracers [^15^O]H_2_O and [^11^C]TMSX were synthesized according to standard operating procedures of the Turku PET Centre as previously described [18, 23]. A combined PET/CT scanner (GE Discovery STE16, General Electric Medical Systems) was used and the subject lay in a supine position with the cervicothoracic area in the axial field of view. CT scans were used for anatomical reference and attenuation correction.

#### [^11^C]TMSX imaging of A_2A_ receptors

To investigate adenosine A_2A_ receptors in BAT, WAT and muscle, PET imaging with A2AR PET radioligand [^11^C]TMSX ([7-*methyl*-11C]-(*E*)-8-(3,4,5-trimethoxystyryl)-1,3,7-trimethylxanthine) was performed on both days with an identical imaging protocol at baseline and in cold conditions. A cannula was placed in the antecubital vein of one arm for administration of radioligands and an arterial cannula was placed in the radial artery of the contralateral arm for blood sampling. After a CT scan of the neck region, [^11^C]TMSX was injected intravenously (baseline 454 ± 65 MBq, cold 478 ± 36 MBq) and dynamic PET images were acquired for 60 min (with frames 6 × 10 s, 3 × 30 s, 5 × 60 s, 5 × 150 s, 8 × 300 s). Arterial blood samples were acquired during the scan for measurement of radioactivity and metabolite analyses. Samples were protected from light, and radioactivity in the plasma was measured using an automatic gamma counter (Wizard 1480 3; Perkin Elmer, Turku, Finland). Fractions of unchanged radioligand and radioactive metabolites in the plasma were measured using high-performance liquid chromatography (HPLC), and the empirical Hill-type function f(t) = p1 – (p1*t)/(p2 + t) was fitted to the fractions of unchanged radioligand. (For details, see Supplemental Material). Due to technical problems, data from one scan could not be acquired, and arterial blood sampling was not possible for two scans.

Carimas 2.9 software (Turku PET Centre, Turku, Finland) was used to analyze PET images. Regions of interest (ROIs) of adipose tissue were drawn manually on fused PET/CT images, including only voxels with CT Hounsfield units within the adipose tissue range (−50 to −250 HU) [24]. BAT ROIs were drawn bilaterally in supraclavicular adipose tissue depots, and WAT ROIs were drawn in subcutaneous areas of the arms. ROIs of skeletal muscle were drawn bilaterally in the deltoid muscle. Regional time-activity curves (TACs) of these tissues were calculated from the dynamic PET images.

To form an accurate TAC of the plasma, we combined an image-derived TAC (obtained from the aortic arch of the PET image) from the first 5 min and the manual arterial plasma samples. The image-derived blood curve was converted to plasma using individual hematocrit values, assuming that radioactivity concentration in blood cells is zero. The plasma TAC was then corrected for the fraction of unchanged radioligand. This method was compared with the standard procedure of using only arterial blood samples as input (see Supplemental Material). Arterial cannulation is an invasive procedure which is demanding for the study subjects and requires resources and the expertise of an anesthesiologist. We therefore also analyzed the [^11^C]TMSX images using strictly an image-derived input function and compared it to the standard analysis method (see Supplemental Material).

Using the regional TACs and metabolite-corrected TAC of plasma, the distribution volume (DV) for [^11^C]TMSX in the BAT, WAT and muscle was calculated using multiple-time graphical analysis for reversible tracer uptake (“Logan plot”, [25]). The start time for line fit was set to 15 min. Examples of Logan plots are shown in the Supplemental Material.

#### [^15^O]H_2_O imaging

[^15^O]H_2_O-PET/CT scans were performed to quantify perfusion of BAT, WAT and muscle. After a CT scan, one bolus of [^15^O]H_2_O (922 ± 56 MBq) was injected intravenously, and a dynamic PET scan was acquired for 5 min (with frames 6 × 5 s, 6 × 15 s, 6 × 30 s). After radioactive decay, a second [^15^O]H_2_O bolus (903 ± 61 MBq) was injected and an identical scan was done with simultaneous intravenous infusion of adenosine (0.14 mg/kg/min). Electrocardiography, heart rate, blood pressure and the well-being of the study subject were monitored during the 5 min of adenosine infusion. On the second day of imaging, an identical scanning protocol with one bolus of [^15^O]H_2_O (945 ± 85 MBq) was performed during controlled cold exposure (See Fig. 1).

Carimas 2.9 software was used for image analysis, and the identical ROIs were used as in the [^11^C]TMSX-PET/CT images described above. An image-derived TAC was obtained from the aortic arch of the PET image and used as the arterial input function. Perfusion in BAT, WAT and muscle was then calculated using the one-tissue compartment model as previously described [10].

### Analysis of blood values

Plasma noradrenaline (NA) values at baseline and after cold exposure were measured in the laboratory of Eastern Finland (ISLAB, Kuopio, Finland) with HPLC. Serum free fatty acid (FFA) and triglyceride values were measured using the enzymatic colorimetric method at the Turku University Hospital laboratory (TYKSLAB, Turku, Finland; for details, see the Supplemental Material).

### Statistical analyses

Statistical analysis was performed with JMP Pro 12 software. Paired *t* tests were used to compare differences between study conditions. Results are expressed as mean ± S.D. and a two-tailed *P* value of <0.05 was considered statistically significant.

## Results

### Adenosine increases perfusion of human BAT, even more than cold exposure

**Fig. 2:**
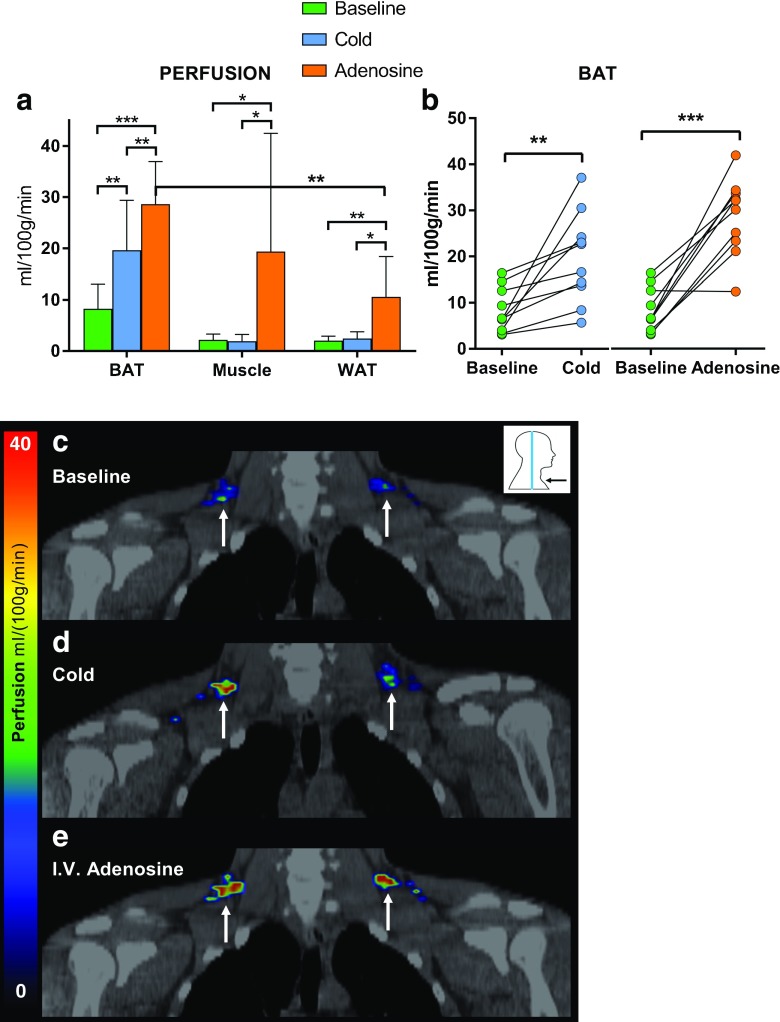
**A**. Perfusion of BAT, muscle and WAT (n = 10) at baseline, during cold exposure and during adenosine administration. **B**. Perfusion in BAT, depicting individual change from baseline to cold, and from baseline to adenosine stimulation. *C*.–*E*. Coronal [^15^O]H_2_O-PET/CT images of BAT from one study subject in all three study conditions. *P < 0.05, **P < 0.01, ***P < 0.001

Cold exposure caused a 2.4-fold increase in BAT perfusion compared to baseline conditions (basal 8.3 ± 4.5 vs. cold 19.6 ± 9.3, *P* = 0.006), while no significant changes were observed in WAT or muscle. Compared to cold, intravenous adenosine markedly increased BAT perfusion (cold 19.6 ± 9.3 vs. adenosine 28.6 ± 7.9, *P* = 0.003). Intravenous adenosine also significantly increased perfusion of muscle (2.2 ± 1.1 vs. 19.4 ± 21.9, *P* = 0.04) and WAT (2.1 ± 0.8 vs. 10.6 ± 7.4, *P* = 0.004). Thereby, perfusion in BAT was 2.7-fold compared to perfusion in WAT during adenosine administration (see Fig. 2).

### Cold exposure decreases A_2A_ receptor availability in BAT

**Fig. 3:**
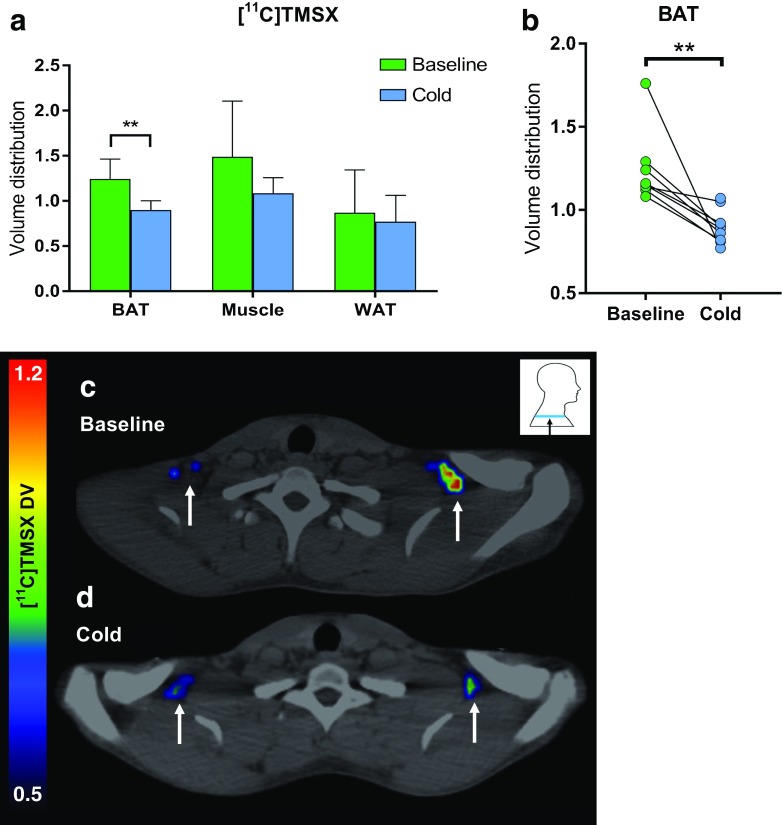
**A**. [^11^C]TMSX distribution volume (DV) of BAT, muscle and WAT (*n* = 8) at baseline and during cold exposure. **P < 0.01. **B**. [^11^C]TMSX distribution volume in BAT, depicting individual change from baseline to cold. **C**.–**D**. Transverse [^11^C]TMSX-PET/CT images of BAT from one study subject in both study conditions

DV of [^11^C]TMSX, the parameter for A_2A_ receptor density, was significantly lower in BAT during cold conditions compared to baseline conditions (cold 0.90 ± 0.10 vs. baseline 1.24 ± 0.21, *P* = 0.006). However, DV of [^11^C]TMSX in muscle or WAT did not change during cold exposure (see Fig. 3). [^11^C]TMSX binding in BAT had a negative correlation with BAT perfusion (*R* = −0.53, *P* = 0.029).

### Systemic noradrenaline levels, free fatty acids and energy expenditure coincide with changes in perfusion and A2AR density of BAT

Compared to room temperature, cold exposure increased plasma NA concentration (2.06 ± 0.65 vs. 5.06 ± 1.42, *P* < 0.0001, Fig. 1B). Additionally, during cold exposure, the plasma levels of FFAs and triglycerides, as well as whole-body EE and the utilization of lipids as an energy substrate all increased (Fig. 1C–F). BAT perfusion was doubled concurrently with the increases in EE, consumption of lipids as energy substrate and plasma FFA levels, while [^11^C]TMSX binding in BAT was simultaneously decreased.

## Discussion

The results of this study show for first time that adenosine is a potent stimulator of human BAT perfusion. Although human BAT perfusion in vivo has previously been studied in some physiological conditions [10–12, 26], the direct effects of adenosine on BAT have not been previously assessed. We found that administration of adenosine caused maximal perfusion of supraclavicular BAT, and the response was even higher than that seen during cold exposure.

Adenosine is known to be a vasodilator, and in line with that, an enhancement of blood flow was observed in muscle and WAT. It is noteworthy, however, that during adenosine administration, BAT perfusion was nearly 3-fold compared to WAT perfusion. BAT is a densely vascularized tissue, and adenosine can bind to A2AR on the vascular smooth muscle cells, resulting in relaxation and vasodilation [27]. Nevertheless, we and others have shown that BAT perfusion is linearly associated with both the metabolic rate of oxygen and the EE of the tissue [10–12]. There is also preclinical evidence that agonism and blockade of A2AR increases and decreases oxygen consumption compared to the vehicle, respectively [8]. Furthermore, it has been shown that adenosine enhances lipolysis and increases expression of thermogenic markers in BAT [8], both being indicative of BAT activation. Taken together, the observed increase in BAT perfusion during adenosine infusion is likely a combined effect of increased vasodilation and BAT activation. The specific molecular mechanisms should be explored further.

A_2A_ receptor agonist treatment in mice has also been shown to increase the function of BAT measured as its glucose uptake [8]. Glucose uptake measurements with [^18^F]FDG-PET imaging were not feasible in this human study due to the rapid breakdown of intravenous adenosine and the short infusion time used to minimize cardiovascular side effects and discomfort of the study subjects. However, with [^15^O]H_2_O-PET imaging, the acute adenosine response can be quantified, and increased perfusion is a marker of BAT metabolic activity and particularly oxygen consumption [10, 11].

We also showed changes in human BAT A_2A_ receptors during cold exposure when BAT is physiologically activated. DV of the A2AR radioligand [^11^C]TMSX decreased significantly in BAT during cold exposure, a response which was not observed in WAT or muscle. [^11^C]TMSX binds specifically to A2AR, and DV quantifies the density of A2AR available for radioligand binding in tissue [28, 29]. During cold exposure, BAT thermogenesis is activated via the sympathetic nervous system (SNS), when NA and its co-transmitter adenosine triphosphate (ATP) are released at efferent nerve endings. ATP is quickly degraded into adenosine, which exerts its effects on BAT locally [6]. This local increased concentration of adenosine during SNS stimulation has previously been measured in BAT of rodents [8]. The decreased binding of [^11^C]TMSX in BAT during cold exposure indicates higher binding of endogenous adenosine, which competes for the same receptors as the radioligand. Similar physiological changes in DV of [^11^C]TMSX have been reported in the skeletal muscle of mice, where a decrease in radioligand binding resulted from higher endogenous adenosine release during exercise [19]. Interestingly, lower binding of [^11^C]TMSX in BAT was associated with increased BAT perfusion, further suggesting that endogenous release of adenosine is accompanied by higher oxidative metabolism of BAT. The observed changes in this study highlight that adenosine and A2AR are significant in cold-induced BAT activation.

To our knowledge, this is the first in vivo human study measuring A2AR density in BAT or WAT. Interestingly, already at baseline conditions we observed a tendency of lower A2AR density in WAT than in BAT (WAT 0.87 ± 0.44 vs. BAT 1.24 ± 0.21, *P* = 0.08); however, the variance of [^11^C]TMSX binding in WAT was high. The low DV in WAT is likely a combination of a small A2AR density and non-specific uptake of the lipophilic tracer, and these factors together result in increased scatter of the PET signal. Future receptor blocking studies are warranted to determine the degree of non-specific binding of [^11^C]TMSX in adipose tissue. Furthermore, the study subjects were all lean young men with only a small amount of subcutaneous WAT in the cervicothoracic area, which may also explain high variance in the WAT PET results. However, the most abundantly expressed adenosine receptors in WAT are the A_1_ receptors, not A_2A_ receptors as in BAT [8, 30]. Studies with overweight subjects or PET scans of the abdominal area are needed to further assess the question of adenosine receptors in WAT. In addition, this was a pilot study with only 10 male subjects; therefore, future research should also include female subjects in order to apply the findings of BAT regulation to a broader population.

BAT and WAT have not been studied with [^11^C]TMSX-PET previously, but a study analyzing A2AR density in human muscle tissue [21] reported similar DV values in skeletal muscle as our current study. The analysis of [^11^C]TMSX PET images has previously required arterial blood sampling for determination of the input function [18, 20, 21]. Arterial cannulation, however, is an invasive procedure which is not always possible or successful, and it requires experienced staff and resources of an anesthesiologist, which may limit the use of the radioligand. Here, we combined an image-derived input curve from the aortic arch with manual arterial blood samples from later time points, and obtained fully comparable results compared to the standard input method (see Supplemental Material). We further tested whether the input function could be derived entirely from the image, thus eliminating the need for any arterial cannulation. The main findings were consistent, but the fully image-derived input function underestimated the DV (see Supplemental Material). Our data therefore show that that the combination of an image-derived and arterial input can be used for analysis of [^11^C]TMSX in peripheral tissues, if a suitably large artery is visible in the PET image.

During cold exposure, systemic NA levels in plasma were higher compared to baseline conditions. Systemic adenosine could not be measured from circulating blood, as it exerts its functions locally at a tissue level and is rapidly broken down [31, 32]. However, since adenosine concentration increases in BAT together with increased NA and ATP release [8], systemic NA was used here as an indirect measure of adenosine release. Increased NA levels were measured concurrently with lower [^11^C]TMSX binding in BAT, further signifying that endogenous adenosine was released in BAT and affected radioligand binding during cold exposure. In cold, we also observed increased resting EE and increased use of lipids as energy substrate, together with an increase in FFAs and triglycerides in plasma. These findings are in agreement with previous studies from our group and others [10, 11, 33], demonstrating the activation of the SNS in the study subjects. Higher BAT perfusion, but lower [^11^C]TMSX binding coincided with the increases in EE, FFA and consumption of lipids as energy substrate. These findings support that the SNS, adenosine and A2AR participate in BAT activation in humans.

In conclusion, these novel imaging findings in humans indicate that adenosine is a potent activator of human BAT physiology. During cold exposure, adenosine A_2A_ receptors are occupied with endogenous adenosine. Targeting the adenosine A_2A_ receptors in BAT could provide a potential way to increase BAT function and improve metabolism in humans.

## Electronic supplementary material

ESM 1 (DOCX 574 kb)
