# Supplementary material for: Regulation of human brown adipose tissue by adenosine and A2A receptors – studies with [15O]H2O and [11C]TMSX PET/CT
Source: Eur J Nucl Med Mol Imaging. 2018 Aug 13;46(3):743–50. doi: 10.1007/s00259-018-4120-2 (PMC6351510; doi:10.1007/s00259-018-4120-2)
Supplement: Supplementary file 1 — (DOCX 574 kb) [file 259_2018_4120_MOESM1_ESM.docx]

*Electronic Supplemental Material*

**Regulation of Human Brown Adipose Tissue by Adenosine and A_2A_ receptors – Studies with [^15^O]H_2_O and [^11^C]TMSX PET/CT**

European Journal of Nuclear Medicine and Molecular Imaging

Minna Lahesmaa^1,2^, Vesa Oikonen^1^, Semi Helin^1^, Pauliina Luoto^1^, Mueez U Din^1,2^, Alexander Pfeifer^3^, Pirjo Nuutila^1,4^, Kirsi A. Virtanen^1,2^

1. Turku PET Centre, University of Turku, Turku, Finland
2. Turku PET Centre, Turku University Hospital, Turku, Finland
3. Institute of Pharmacology and Toxicology, University of Bonn, Bonn, Germany
4. Department of Endocrinology, Turku University Hospital, Turku, Finland

Corresponding author:

Kirsi A. Virtanen

E-mail: kirsi.virtanen@utu.fi

Radioactive metabolite measurements of [^11^C]TMSX

Arterial blood samples were manually acquired during the scan at time points 5, 7, 10, 20, 30, 40, 50, and 60 minutes for measurement of total radioactivity concentration in plasma, and at time points 5, 10, 30, and 50 minutes for metabolite analysis. Radioactivity of arterial samples from earlier time points (0-5 min) were measured with an automatic blood sampling system of the Turku PET Centre as previously described [1].

Metabolites of [^11^C]TMSX were measured from arterial plasma at time points 5, 10, 30 and 50 min using high-performance liquid chromatography (HPLC). HPLC consisted of LaChrom Instruments (Hitachi; Merck, Darmstadt, Germany): pump L-7100, UV-detector L-7400; and Interface D-7000**,** of an online radioactivity detector (Radiomatic 150TR, Flow Scintillation Analyzer; Packard, Meriden, CT, USA) and a computerized data acquisition system. Radio-HPLC was performed using a μ-Bondapak® C-18 column (125 Å, 10 µm, 7.8 × 300 mm; Waters, Milford, MA, USA) at a flow rate of 6.0 mL/min and a gradient of acetonitrile (A) and phosphoric acid, 50 mmol/L (B) as follows: 25% A and 75% B at 0 − 2 min, 80% A and 20% B at 6 − 8 min, and 25% A and 75% B at 8.5 − 10 min.

Empirical Hill-type function

The empirical Hill-type function f(t)=p1-(p1*t)/(p2+t) was fitted to the fractions of unchanged radioligand. For the reported results, the Hill-type function was fitted individually to the fraction measurement of each subject. If no individual value was available (due to unsuccessful blood sampling), population mean value was used. Figure 1 shows the collected fits for the fractions from all subjects in a) baseline conditions, b) cold conditions, or c) baseline and cold conditions combined. The fitted parameters were p1=994.0 and p2=0.9866 in baseline; p1=1072 and p2=0.9906 in cold; and p1=1032 and p2=0.9884 in baseline and cold conditions combined. The relative fraction of unchanged radioligand was over 90% throughout the study, and there was no significant difference between baseline and cold conditions.

**
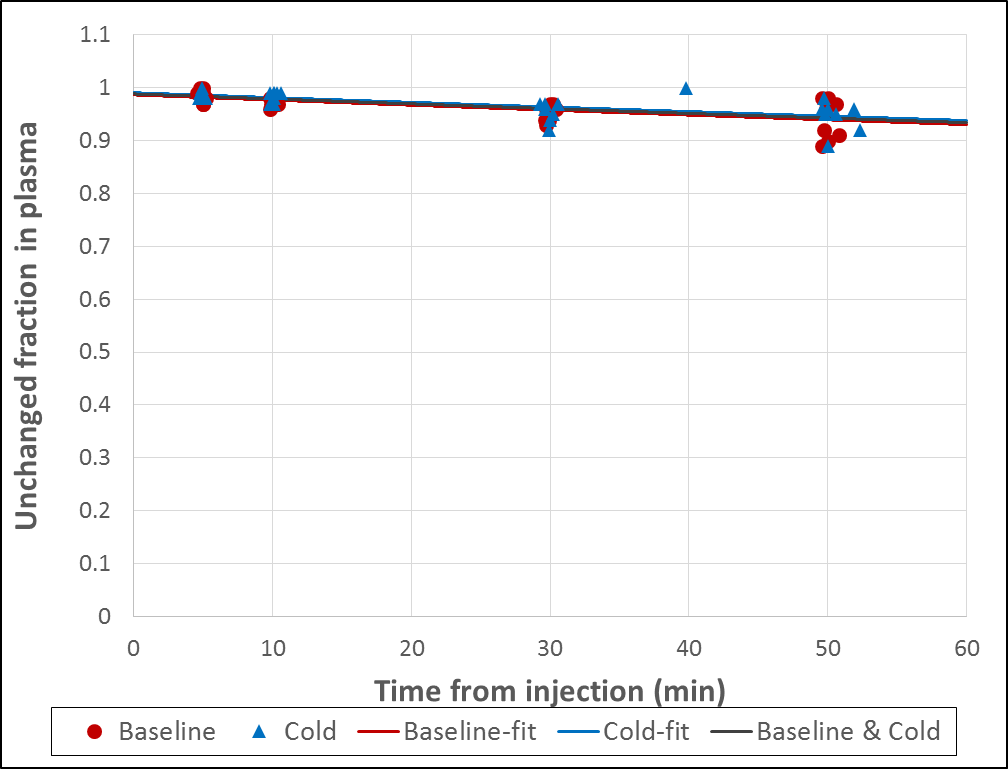
**

**Figure 1. The fractions of unchanged [^11^C]TMSX in plasma as a function of time.**

[^11^C]TMSX contains [^11^C]methyl group, attached to nitrogen, suggesting that [^11^C]CO_2_ and its products, [^11^C]urea, [^11^C]glucose, and [^11^C]lactate would be the main radioactive metabolites. These radioactive metabolites will be taken up by the studied tissues, and probably at different rates during baseline and cold. However, with [^11^C]TMSX the relative fraction of unchanged radioligand is very high, and we therefore assume that DV represents the specific and nonspecific uptake of [^11^C]TMSX.

Comparison of arterial and image derived input functions in [^11^C]TMSX analysis

Distribution volume of [^11^C]TMSX in different tissues is calculated using the time-activity curve of the unchanged radioligand in plasma (input curve) and the regional tissue time-activity curve. Previously, the input curve has been determined from arterial blood sampling [1,2]. This method is invasive for the study subject, sampling is not always successful, and radioactivity measurements at early time points are subject to error. We therefore calculated image-derived input curves and compared them with the standard arterial method.

**Input A =** fully arterial blood sampling (current standard)

**Input B =** combined image-derived input (time points 0-5 min) with arterial blood sampling (time points 5-60 min)

**Input C =** fully image-derived input, no blood sampling (metabolite correction based on population average)

**
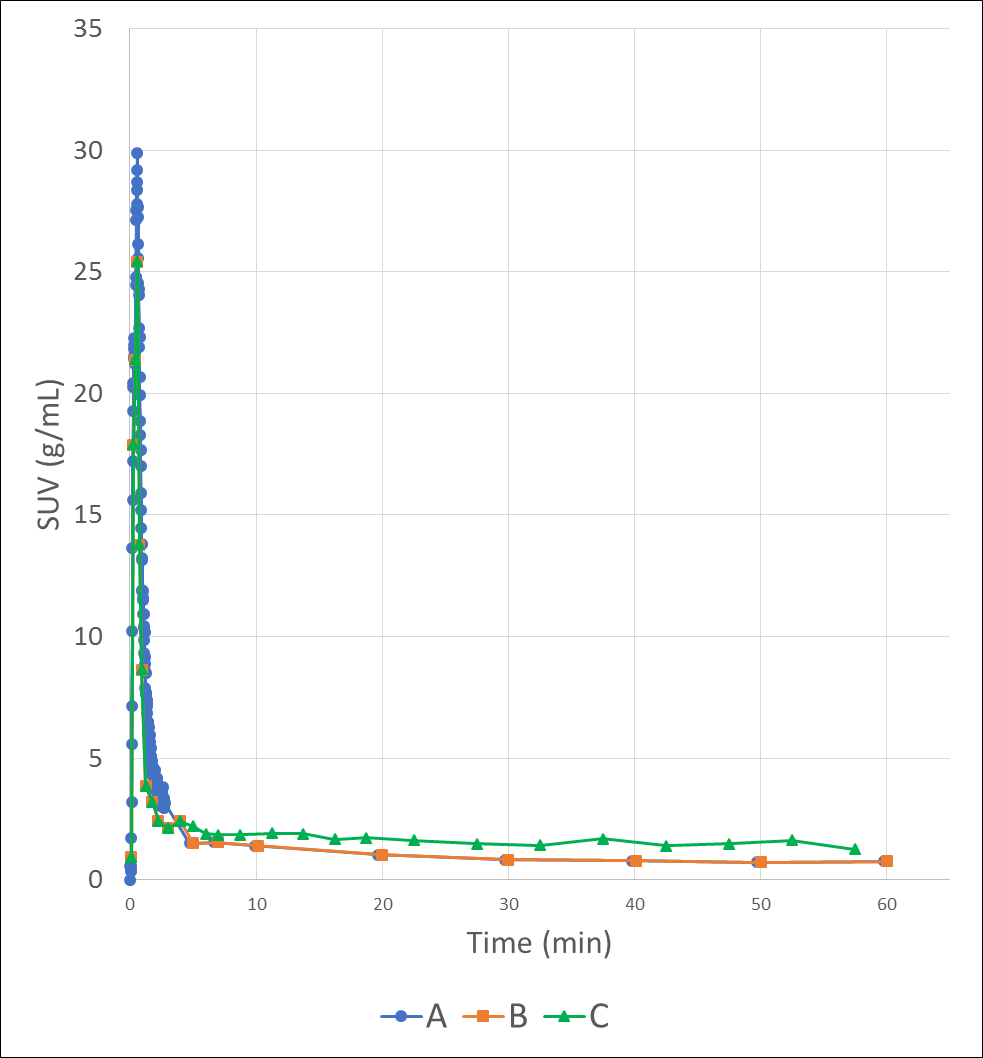
Figure 2. Comparison of the different input functions of one study subject. The first minutes of the different curves are well comparable. At later time points, input C remains at a slightly higher level than inputs A and B.**

**
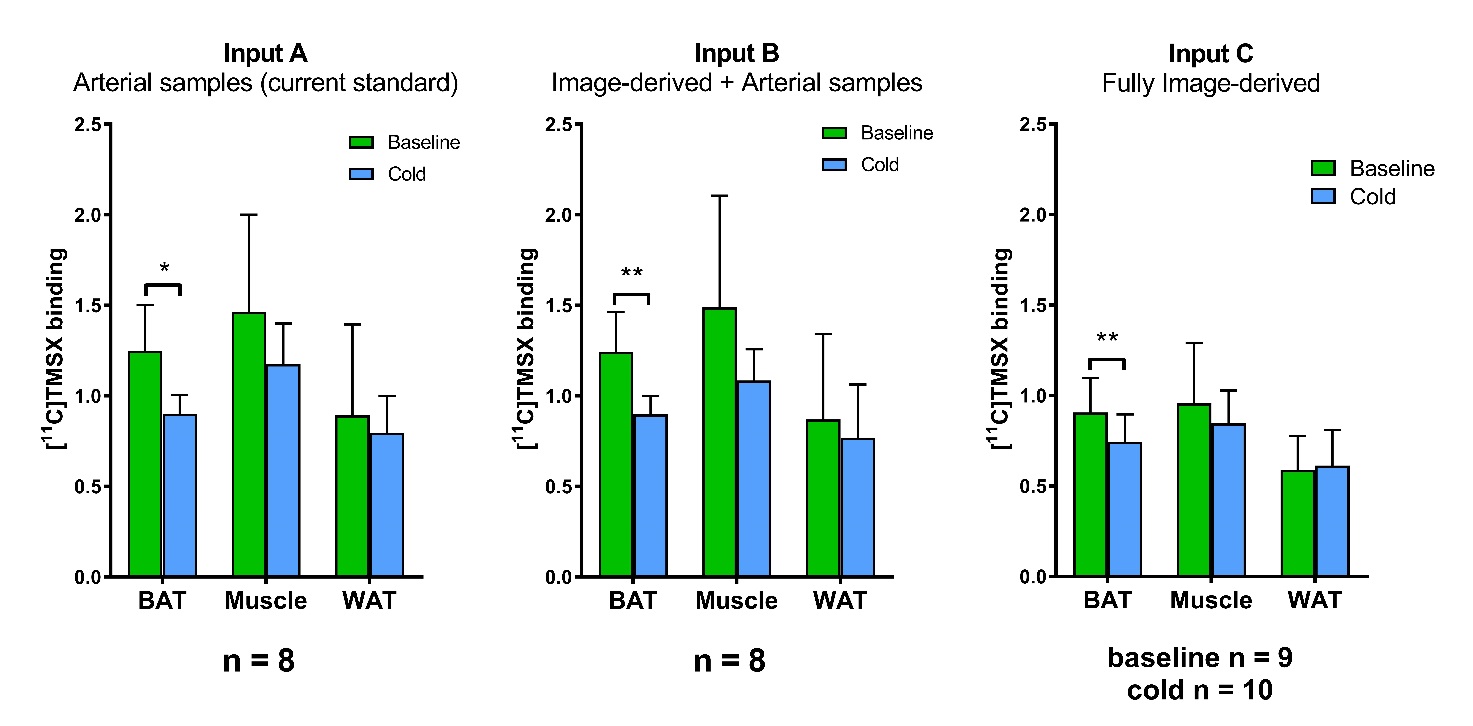
**

**Figure 3. Comparison of distribution volume (DV) in brown adipose tissue (BAT), muscle and white adipose tissue (WAT) using the different input functions.**

**
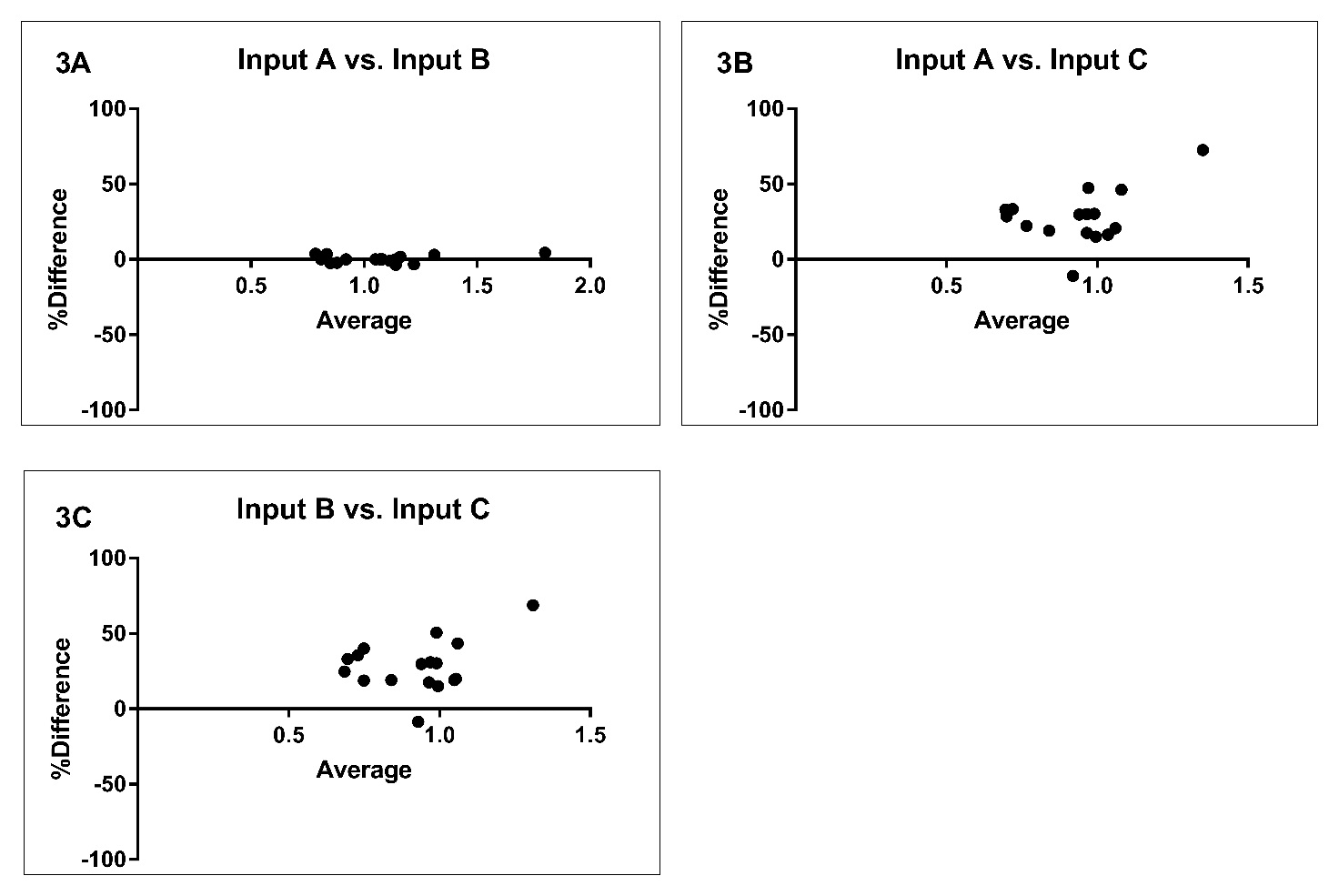
**

4C

4B

4A

**Figure 4. Bland-Altman plots comparing the DV measurements in BAT using different input functions.**

**4A) standard arterial input [A] compared to the combined image-derived and arterial samples [B];**

**4B) standard arterial input [A] compared to the fully image-derived input [C];**

**4C) combined input [B] compared to the fully image-derived input [C]**.

Figure 2 depicts that input curves A and B are well comparable, but input C remains slightly higher at later time points. The Bland-Altman plot analysis is an accurate way to evaluate bias between two methods of measurement [3]. As seen from Figures 3 and 4, using the inputs A and B we obtain fully comparable results. Hence the beginning of the input function can be obtained from the aortic arch of the PET image, and combined with arterial blood samples. Furthermore, a fully image-derived input curve (input C) gives the same findings of the study, but DV values are underestimated. This is likely due to spill-in radioactivity from surrounding tissues, which overestimates the input curve causing underestimation of the DV values.

A fully image-derived input curve is therefore not recommended, if obtaining quantitative results is needed. However, in our comparative research frame in this study, a fully image-derived input gave the same findings as the inputs with arterial samples. There are methods found in literature of PET image analysis which could be used to correct the image-derived input and make it quantitatively equivalent to the arterial input. These methods require testing and further validation for [^11^C]TMSX. We conclude however, that a combination of an image-derived and arterial input can be used for analysis of [^11^C]TMSX, if a suitably large artery is visible in the PET image.

Examples of Logan Plots

DV for [^11^C]TMSX in BAT, WAT, and muscle was calculated using multiple-time graphical analysis for reversible tracer uptake, known as the Logan plot [4].

**
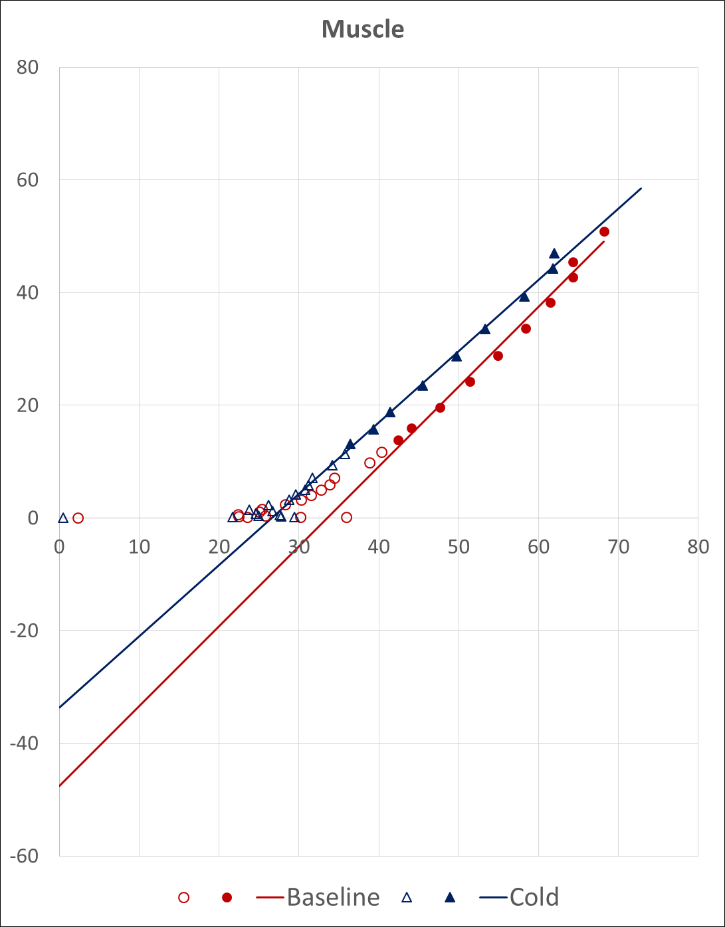
**
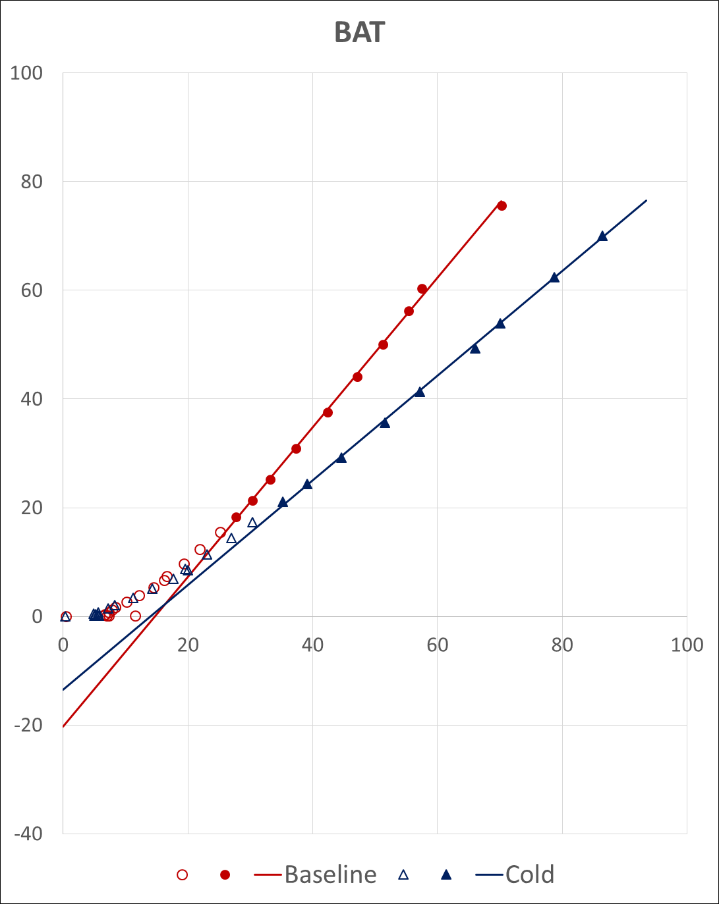

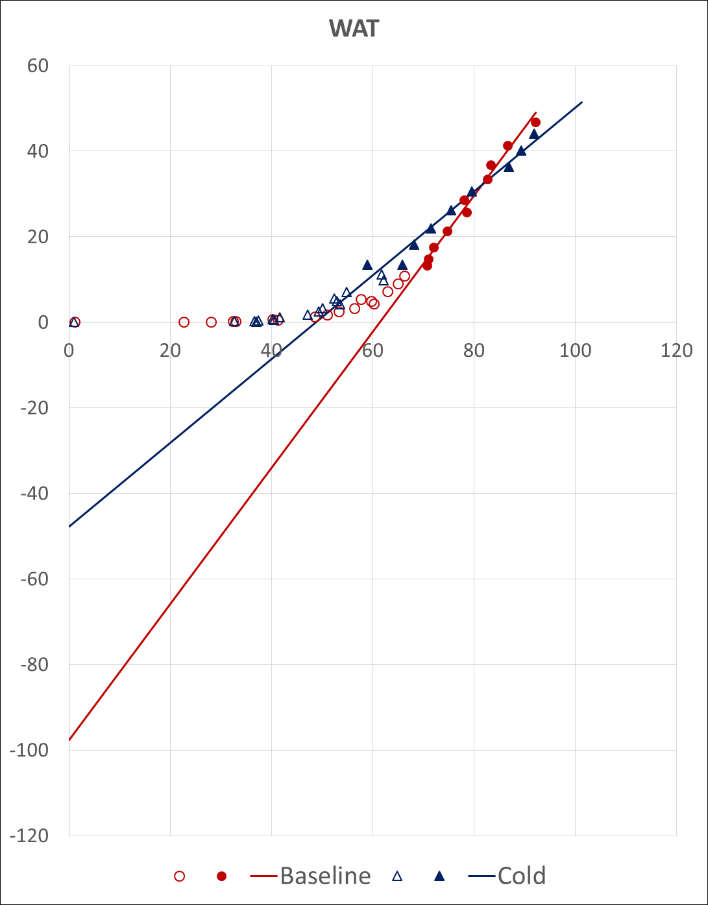


**Figure 5. Representative Logan plots from one subject in baseline and cold conditions. The line was fitted to the data points collected between 15 and 60 min post injection (solid symbols); open symbols represent data points collected before 15 min. The slope of the line equals distribution volume.**

Blood analysis of noradrenaline, free fatty acids and triglycerides

Plasma noradrenaline values at baseline and after cold exposure were measured in the laboratory of Eastern Finland (ISLAB, Kuopio, Finland) with high-performance liquid chromatography (HPLC). A Chromsystems reagent kit (Chromsystems Instruments and Chemicals GmbH, Munich, Germany) was used. Chromsystems Plasma Endocrine Controls, level I (#0010) and level II (#0020) were used to control the performance of the method. The chromatographic system consisted of Agilent 1260 Infinity Quaternary Pump, Agilent 1260 Infinity Autosampler with Agilent 1290 Thermostat (Agilent Technologies, Waldbronn, Germany), Chromsystems #5100 HPLC column for plasma catecholamines (Chromsystems), and Gilson ESA, Coulochem III detector, equipped with model 5020 Guard Cell and 5011A Analytical Cell (ESA, Bedford, MA, USA). The data were analyzed by Agilent ChemStation chromatography program.

Serum free fatty acid (FFA) and triglyceride values were measured at baseline and after cold exposure using the enzymatic colorimetric method at the Turku University Hospital laboratory (TYKSLAB, Turku, Finland). Specifically, FFA were determined using the ACS-ACOD Method (Wako Chemicals GmbH, Neuss, Germany). A Cobas 8000 c502 Analyzer (Roche Diagnostics GmbH, Mannheim, Germany) was used for both FFA and triglyceride analysis.

Supplementary References

1. Rissanen E, Virta JR, Paavilainen T, Tuisku J, Helin S, Luoto P, et al. Adenosine A2A Receptors in Secondary Progressive Multiple Sclerosis: A [11C]TMSX Brain PET Study. J. Cereb. Blood Flow Metab. 2013;33:1394–401.

2. Heinonen I, Nesterov S V, Liukko K, Kemppainen J, NÃ¥gren K, Luotolahti M, et al. Myocardial blood flow and adenosine A2A receptor density in endurance athletes and untrained men. J. Physiol. 2008;586:5193–202.

3. Bland JM, Altman DG. Statistical methods for assessing agreement between two methods of clinical measurement. Lancet (London, England). 1986;1:307–10.

4. Logan J, Fowler JS, Volkow ND, Wolf AP, Dewey SL, Schlyer DJ, et al. Graphical Analysis of Reversible Radioligand Binding from Time—Activity Measurements Applied to [ *N* - ^11^ C-Methyl]-(−)-Cocaine PET Studies in Human Subjects. J. Cereb. Blood Flow Metab. 1990;10:740–7.
